# The associations between intimate partner violence and maternal health care service utilization: a systematic review and meta-analysis

**DOI:** 10.1186/s12905-019-0735-0

**Published:** 2019-02-26

**Authors:** Abdulbasit Musa, Catherine Chojenta, Ayele Geleto, Deborah Loxton

**Affiliations:** 1 College of Health and Medical Sciences, Haramaya University, Harar, Ethiopia; 2 Research Centre for Generational Health and Ageing, Faculty of Health and Medicine, University of Newcastle, Newcastle, Australia

**Keywords:** Systematic review, Meta-analysis, Intimate partner violence, Maternal health care service use

## Abstract

**Background:**

Intimate partner violence exposes women to a wide range of health problems that can either directly or indirectly lead to maternal death. Although in a number of studies intimate partner violence has been associated with inadequate utilization of antenatal care and skilled delivery care, in other studies no association has been found. Therefore, we aimed to comprehensively review the evidence, and quantify the strength and direction of the association between intimate partner violence and utilizing adequate antenatal and skilled delivery care services.

**Method:**

We systematically searched studies from MEDLINE, Embase, Psych INFO, CINAHL, and Maternity and Infant Care. Two independent reviewers screened the articles for eligibility. Quality and risk of bias in the articles were evaluated by using the Newcastle-Ottawa scale for observational studies. Pooled odds ratios and 95% confidence intervals were computed to estimate the association of intimate partner violence and antenatal care, and skilled delivery care. Random-effects models were used to allow for the significant heterogeneity that might possibly be found between studies. The degree of heterogeneity was expressed by using the I^2^ statistic.

**Results:**

The meta-analyses have shown that women who experienced intimate partner violence had 25% decreased odds (AOR = 0.75, 95%CI = 0.61, 0.92) of using adequate antenatal care than those who did not experience IPV. Similarly, women who experienced IPV had 20% decreased odds (AOR = 0.8, 95%CI = 0.69, 0.92) of using skilled delivery care compared to those who did not experience IPV.

**Conclusion:**

The meta-analyses indicated that experiencing intimate partner violence is associated with a lower likelihood of receiving adequate antenatal care and skilled delivery care. Both community-based and facility-based interventions that target the reduction of partner violence, and strictly implementing proven health facility-based counselling interventions, could aid in improving utilization of maternal health care services.

**Electronic supplementary material:**

The online version of this article (10.1186/s12905-019-0735-0) contains supplementary material, which is available to authorized users.

## Background

Maternal mortality continues to be a public health problem worldwide. The World Health Organization (WHO) has indicated that, in 2015 alone, over 303,000 maternal deaths resulting from pregnancy and delivery related complications were reported globally [1]. To reduce maternal mortality, WHO recommends all pregnant women receive antenatal care and skilled delivery care [2, 3].

Antenatal care can be defined as the care provided by skilled health care professionals to pregnant women in order to ensure the best health conditions for both mother and baby during pregnancy [4]. Similarly, skilled delivery care is defined as the care provided to women during labour and delivery by professionals who have been educated and trained with midwifery skills [5].

Affecting one in three ever-partnered women worldwide in their life time [6], Intimate Partner Violence (IPV) exposes women to a wide range of health problems that can either directly or indirectly lead to maternal death [7]. A substantial amount of research across the world has indicated a negative association of IPV with the uptake of adequate antenatal care, and skilled delivery care [8–11].

Although in a number of studies, researchers have reported an association between IPV and inadequate antenatal care [8, 9, 11, 12], and low utilization of skilled delivery care [10, 11, 13], in other studies no evidence of such an association has been found [14, 15]. The methodological quality of the studies, IPV assessment tools, and outcome measurement disparity among studies might contribute to this disparity [16, 17].

Given this inconsistency in the findings, it is important to review the existing evidence to determine the relationship between IPV and maternal health care services (antenatal care, and skilled delivery care) utilization. Although a previous systematic review has explored the relationship between IPV and maternal health care service use, including utilization of antenatal care and facility delivery [18], it did not specifically address the adequacy of antenatal care service and skilled delivery care. Thus, this study will comprehensively review the evidence, and quantify the strength and direction of the association between IPV and antenatal care adequacy and skilled delivery care utilization.

The aim of the study was to answer the question of whether exposure to IPV is associated with inadequate antenatal care utilization among ever-pregnant women, and whether IPV exposure is associated with not utilizing skilled delivery care among women who have ever given birth.

## Method

Registration: This systematic review and meta-analysis was registered on Prospero with the registration number CRD42017075543.

### Eligibility criteria

Any peer reviewed observational studies (cohort, case–control, and cross-sectional studies) that assessed the association of IPV with antenatal care and skilled delivery care were included in this study. Observational studies that did not report the association of IPV with outcome variables by controlling possible confounders were excluded from the study. The review was not restricted by study setting or year of publication.

### Exposure and outcomes

This paper uses the WHO definition of IPV. According to WHO, IPV is defined as “any behaviour within an intimate relationship that causes physical, psychological or sexual harm to those in the relationship. Such behaviour includes acts of physical aggression, such as slapping, hitting, kicking and beating, as well as psychological abuse, such as intimidation, constant belittling and humiliation, and forced intercourse and other forms of sexual coercion. IPV can also include various controlling behaviours, such as isolating a person from their family and friends, monitoring their movements, and restricting their access to information or assistance” [19]. Therefore, the exposure for this study was women who reported an experience of at least one aspect of IPV, whether physical, sexual, emotional or control. Women with no history of any aspects of IPV were taken as the comparator group.

The outcomes were antenatal care adequacy and skilled delivery care utilization. The 2002 WHO definition utilised by each of the studies included in the review was used to define antenatal care adequacy, where antenatal care services were considered adequate if women received four or more visits during pregnancy [16]. Skilled delivery care utilization was defined as having occurred if women received assistance during labour and delivery by a health professional with midwifery skills [2].

### Search method

A comprehensive review of English language literature using the databases OVID MEDLINE, OVID Embase, OVID Psych INFO, OVID CINAHL, and OVID Maternity and Infant Care was performed. The searches were carried out from the inception of each database up to 05/09/2017. Search strategies were tailored to each database to employ the correct search terms. Where possible, both MeSH and free text terms with synonyms were used to increase identification of relevant studies. The following search terms were used to search for the available literature: (intimate partner violence OR partner abuse OR spouse abuse OR partner violence OR battered women OR domestic violence) AND (maternal health service OR maternal care service, OR antenatal care OR ANC OR prenatal care OR PNC OR pregnancy, OR pregnant women OR skilled birth attendant OR institutional delivery OR delivery at health facility). The search terms are available as Additional file 1.

### Screening and selection procedure

Two independent reviewers (AM, AG) screened the articles. First, the titles and abstracts of articles were screened to identify whether the articles were eligible for full text screening. Then, the two reviewers critically examined the full text of the articles based on the study eligibility criteria. Whenever there was a disagreement as to which article was to be included for full title and abstract screening as well as for full paper review, this was resolved through discussion.

### Data collection process

Two reviewers (AM, AG) independently extracted the data from eligible articles. The Joanna Briggs Institute (JBI) data extraction tool for observational studies was used to extract the data. The following variables were extracted: authors, year of publication, sample size, study design, study settings, types of violence, IPV assessment tools, IPV exposure period, main outcomes of the study, adjusted odds ratio of each outcome and confounder adjusted for the outcome.

### Quality and risk of bias assessment

Quality and risk of bias in the articles were evaluated by using the Newcastle-Ottawa Scale (NOS) [20] for observational studies. Two reviewers (AM, AG) independently assessed the quality of each primary article. Any discrepancy in rating the quality was resolved through discussion. A system of points (stars) was given to the eligible categories. Since all studies included in the analysis were cross-sectional, the NOS with a total scale of six was used. A total NOS score of four or above out of six, as used by other studies [21, 22] was used to categorize articles as high quality.

### Strategy for data synthesis

The individual studies were described using summary tables. The analysis was conducted using ProMeta version 3.0 software. Pooled odds ratios with 95% confidence intervals were computed to estimate the association of IPV with antenatal care adequacy and skilled delivery care utilization. Random-effects models were used to allow for the significant heterogeneity that might exist between studies. The degree of heterogeneity was expressed by using the I^2^ statistic. The odds ratio was considered significant if the confidence interval did not include 1.0. Similarly, I^2^ estimates were considered statistically significant at a *P* value of < 0.1. The risk of publication bias was evaluated by using Egger’s test.

Some studies reported multiple estimates using different types of IPV on the same sample of participants. In order to avoid double-counting participants, in studies that reported on more than one aspect of partner violence, preference was given to one estimate that reported on combined IPV (if the study reported on combined IPV). However, in any study with multiple estimates that did not report on combined IPV, preference was given to types of IPV with the most precise estimate (with a narrow confidence interval) as used in the previous study [23].

We also carried out further analysis to precisely establish the relationship between each aspect of IPV and maternal health care services use. In addition, other confounders of IPV that were found to have an association with antenatal care adequacy and skilled delivery care were reviewed and discussed.

## Results

### Literature search

We retrieved 6553 potentially relevant articles, from which 2969 duplicated articles were removed and 3584 were further screened by full title and abstract. Of the articles screened by title and abstract, 3540 did not meet the inclusion criteria and 44 full text articles were further assessed for eligibility. From these, 34 articles did not meet the eligibility criteria.

One reason for excluding these articles was outcome measurement disparities: by which we excluded thirteen articles that measured antenatal care adequacy not in line with the antenatal care adequacy definition used in this paper. In addition, the descriptor ‘not related to outcomes’ was used to exclude eight articles that reported on early booking and the presence /absence of antenatal care, rather than antenatal care adequacy, and two articles that reported on the location of birth rather than skilled delivery care services utilization. Furthermore, other criteria such as review studies, studies that addressed domestic violence (not exclusive to IPV) and non- peer reviewed articles were excluded from the study.

**Fig. 1:**
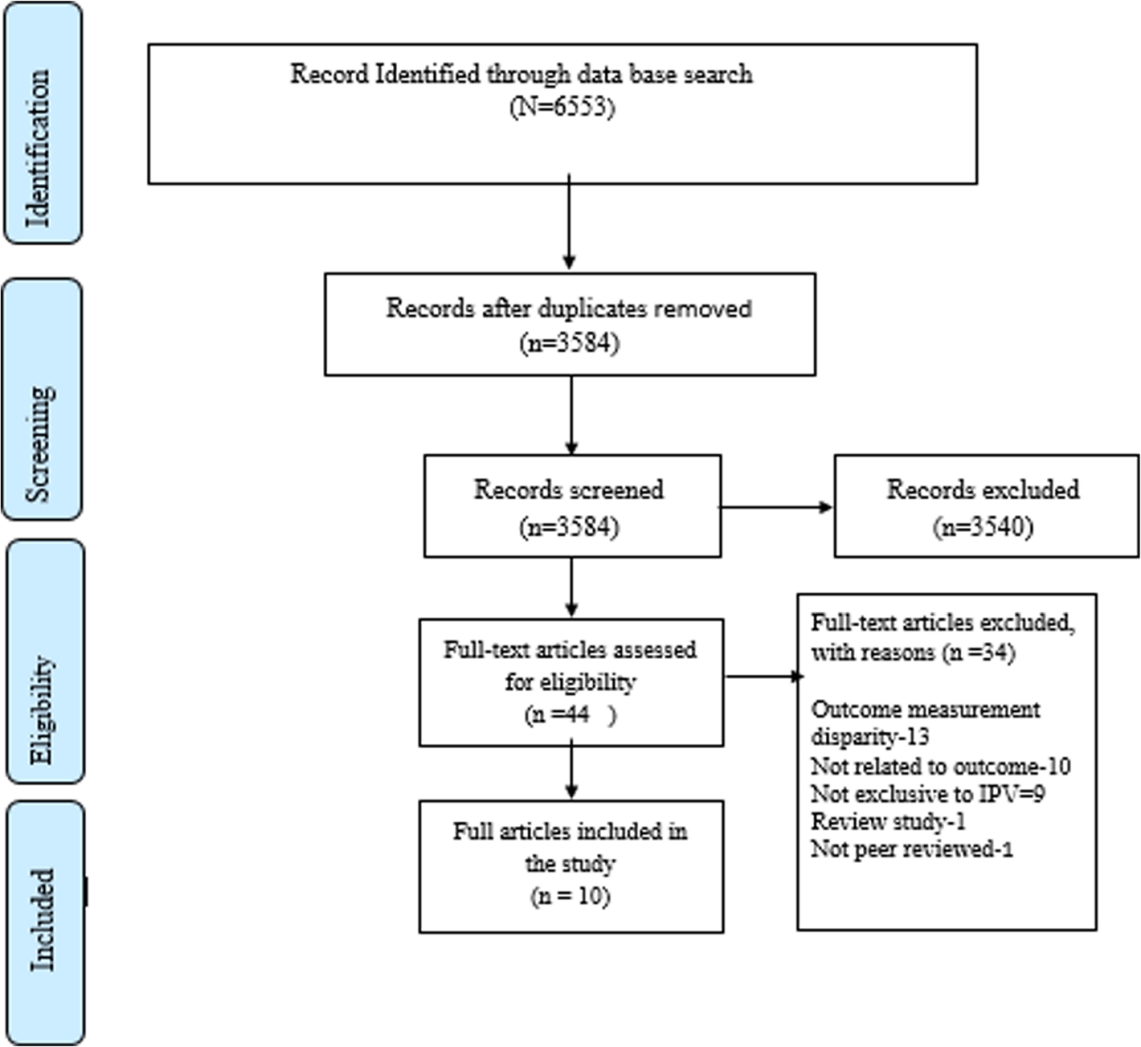
PRISMA flow diagram of study selection

Finally, ten articles that fulfilled the eligibility criteria were included in this systematic review and meta-analysis, while one article [14] that did not report on the odds ratio with a confidence interval was excluded from meta-analysis but included in the systematic review (Fig. 1).

### Characteristics of the study included in the meta-analysis and systematic review of IPV and antenatal care adequacy

Overall, six studies with a total of 16,360 respondents were included for antenatal care adequacy assessment. Among the included studies, four were from Africa [14, 24–26] while the remaining two were from Asia [27, 28]; all studies were community based cross-sectional studies. Regarding types of IPV, two studies reported on combined IPV [27, 28], five studies reported on physical IPV [14, 24–26, 28], two studies reported on sexual IPV [24, 28] while only one study reported on control [24]. Regarding the IPV assessment tool, three studies used Self (with WHO component) tools [14, 25, 26], one study used WHO tool [24], one study used the DHS tool [27] and the remaining study used the conflict tactics scale [28] (Table 1).

**Table 1:** Characteristics of study included in Meta-analysis and Systematic review of IPV and Antenatal care adequacy

Study	Sample size	Study location	Study setting	Types of violence	IPV exposure time	IPV assessment scale	NOS quality Score	Confounders
[24]	210	Ethiopia	Community / Primary survey	Physical, Sexual, emotional, partner control	Ever in current relation	WHO tool	6	women’s age, couple’s age gap, women’s educational status, partner’s educational status, women’s decision-making autonomy, women’s employment status, women’s weekly mass media exposure, couple’s relationship duration and household monthly income
[27]	294	Timor-Leste	Community /Timor-Leste DHS	IPV general	Ever in current relation	DHS tool	4	Age of the women, women’s education, and wealth.
[25]	6871	Nigeria	Community / state-wide survey	Physical IPV	Prior year	Self (With WHO component)	6	Women’s education, house hold head education, Age of the women, residency, help from family member, ownership of motorized transport, marital status/ cohabiting, wealth, proximity to government health facility, information from health worker regarding pregnancy, gravidity
[28]	2001	Bangladesh	Community/Bangladesh DHS	Physical, sexual, combined IPV	Lifetime	Conflict tactic scale	5	Women’s age, women’s education, husband’s education, women’s decision-making and freedom of movement autonomy, women’s occupation, residence, religion, frequency of mass media exposure, parity, pregnancy intentions, and wealth index category
[26]	418	Ghana	Community /Nationally representative data	Emotional and physical violence during pregnancy	Pregnancy	Self with WHO components	5	Women’s age, marital status, women’s education, religion, wealth quantile, residency, general health of the women, region of the respondent, number of the children
[14]	6566	Egypt	Community/Egypt DHS	Physical	Life time	Self	3	Women’s education, residency, parity

Concerning the ascertainment period for IPV, two studies reported on IPV ever experienced in a current relationship [24, 27], two other studies reported on IPV experienced in a lifetime [14, 28], one study reported on IPV experienced during pregnancy [26] while the remaining study reported on IPV experienced in the year prior to the survey being conducted [25].

### Meta-analysis of IPV and antenatal care adequacy

**Fig. 2:**
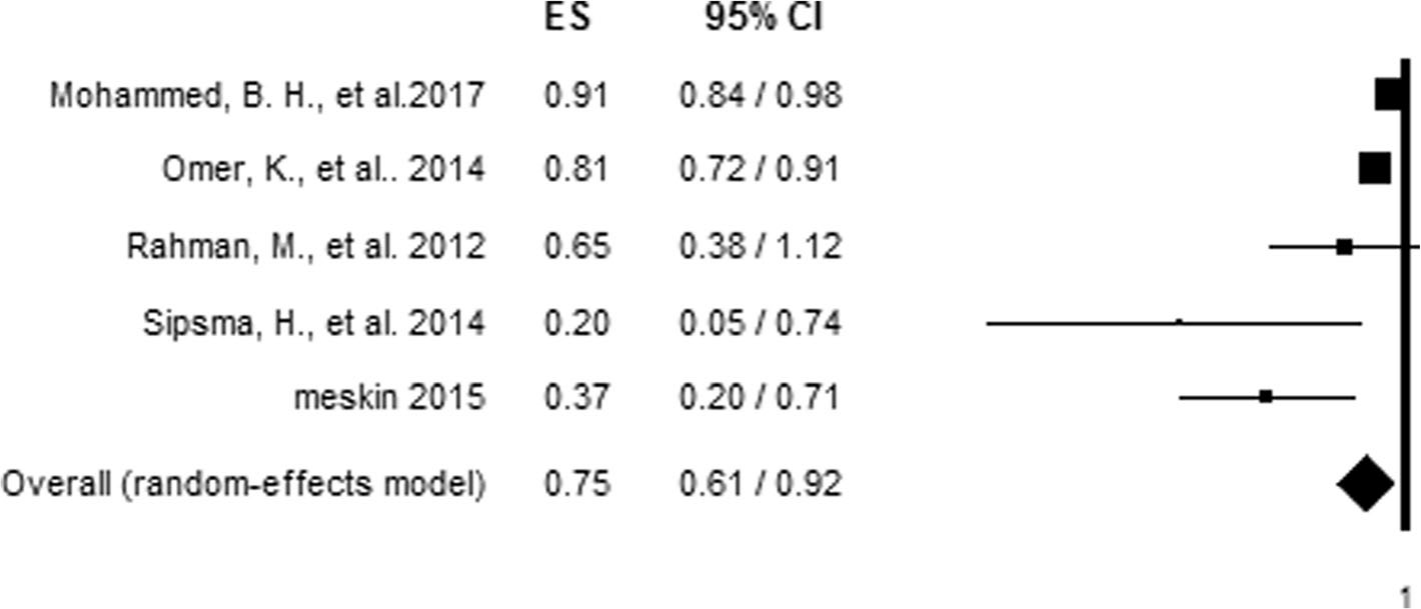
Forest plot of an association between IPV and adequate antenatal care utilization

To evaluate the relationship between IPV and antenatal care adequacy, articles that reported on more than one aspect of IPV in relation to antenatal care adequacy were first made to be represented by a single effect size, giving preference to combined IPV [28] and the effect size with the most precise estimate respectively [24]. The association of each aspect of IPV with antenatal care adequacy was assessed by pooling the effect of each IPV in the analysis. The pooled analysis showed women who experienced IPV had 25% decreased odds (AOR = 0.75, 95%CI = 0.61, 0.92) of using adequate antenatal care than those who did not experience IPV (Fig. 2). However, a significant level of heterogeneity (I^2^ = 74.01%) and possible risk of publication bias (Egger’s test, *P* value = 0.022) were observed among studies included in the analysis (Table 2).

**Table 2:** Hetrogeneity test for analysis of IPV and antenatal care utilization

IPV	Hetrogeniety test				
	X^2^	P-value	I^2^	T^2^	T
Combined	1.76	0.185	43.15	0.07	0.26
Physical	7.26	0.064	58.70	0.05	0.23
Sexual	1.84	0.175	45.63	0.03	0.16
Over all hetrogeniety	15.39	0.004	74.01	0.03	0.16

### Different types of IPV and adequate antenatal care service utilization

**Fig. 3:**
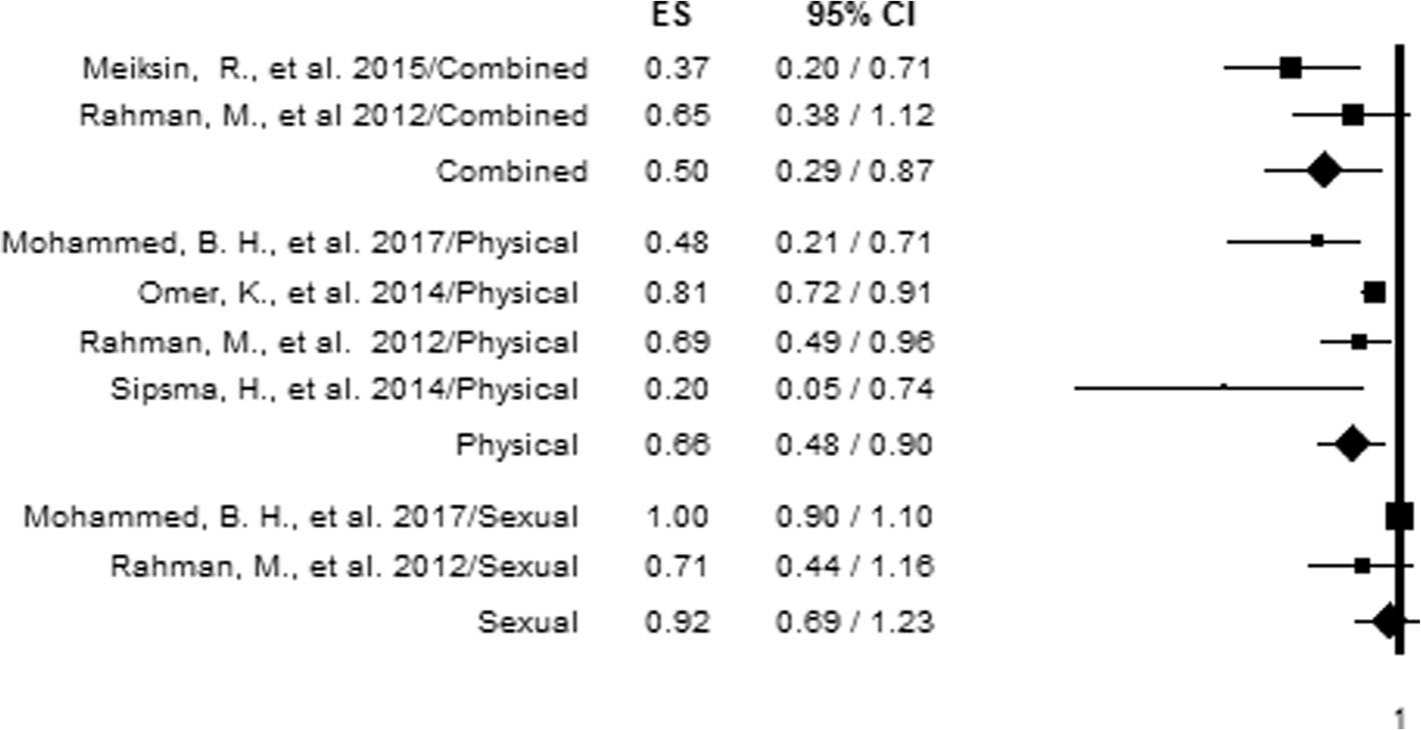
Forest plot of an association between different types of IPV and adequate antenatal care utilization

Due to the existance of studies that reported on more than one aspect of IPV, we also computed further analysis to precisely establish the relationship between each aspect of IPV and antenatal care adequacy. Specific IPV types were included in the analysis if reported in two or more studies. Hence, emotional violence and partner control were excluded from the analysis, since no more than one study investigated their relationship with antenatal care adequacy. From the analysis, experiencing each type of IPV was found to be associated with utilizing adequate antenatal care, with the exception of sexual violence. The analysis indicated women who reported experiencing combined IPV had 50% decreased odds (AOR = 0.50, 95%CI = 0.29, 0.87) of using adequate antenatal care compared to those who did not report experiencing IPV, while those who reported experiencing physical violence had 34% decreased odds (AOR = 0.66, 95%CI = 0.48, 0.90) of using adequate antenatal care (Fig. 3). No significant level of heterogeneity was observed among studies that assessed combined (*P* value = 0.185) and sexual violence (*P*-value = 0.175), while physical IPV indicated significant heterogeneity (I^2^ = 58.70%, with *P*-value of 0.064) (Table 2).

### Factors associated with utilizing adequate antenatal care

According to the articles reviewed, age was found to have an association with receiving adequate antenatal care, indicating that older women are more likely to utilize adequate antenatal care [25, 28]. Similarly, women who received primary and above education [14, 25, 28], whose husbands’ received primary and above education [14, 28] or those with an educated head of household [25] were more likely to utilize adequate antenatal care.

Women who were categorized as rich by the Demographic and Health Survey (DHS) wealth index [28], or who were not living in absolute poverty [25] were more likely to utilize adequate antenatal care. Similarly, women who resided in urban areas [25, 27, 28], who were married/cohabiting [25] and had their own motorized transport [25] were more likely to utilize adequate antenatal care than their counterparts.

In addition, primiparous women [14, 28] were more likely to utilize adequate antenatal care than their counterparts, while having received information from health workers regarding pregnancy was also associated with women’s adequate use of antenatal care [25] (Table 3).

**Table 3:** Covariates associated with adequate antenatal care utilization

Covariate	Variables	Antenatal care adequacy utilization	
		Reported AOR, CI	References
Socio-demographic	Age	15–24 yrs. = Ref, 25–34 yrs. = 1.50,(0.99–2.28), 35–49 yrs. = 2.45 (1.23–4.88)	[28]
		18 years and below = Ref Above 18 yrs. = 1.49 (1.20–1.84)	[25]
	Maternal education	No education = Ref, primary = 1.35 (0.76–2.40), Secondary+ = 3.0 (1.71,5.26)	[28]
		No education = Ref Educated = 1.26 (1.06–1.48)	[25]
		Less than secondary education- Ref, Secondary and above = AOR,2.56, *P* value< 0.05	[14]
	Husband’s Education	No education = Ref Secondary+ = 1.56 (1.03,2.36)	[28]
	Household head education	Uneducated = Ref Educated = 1.16 (1.01,1.34)	[25]
	Wealth index	Poor = Ref Rich = 1.62 (1.01–2.64)	[28]
	Not being poor	No enough food in house = Ref, Has enough food in house hold = 1.20 (1.05–1.37)	[25]
	Residency	Rural = Ref Urban = 2.18 (1.51–3.14).	[28]
		Rural = Ref Urban = 1.54 (1.21–1.96)	[25]
		Urban = Ref Rural = AOR, 0.27, *P*-value< 0.05	[14]
		Urban = Ref Rural = AOR, 0.43 (0.20, 0.91)	[27]
	Marital status	Single = Ref Married/Cohabited = 1.24 (1.04–1.47)	[25]
	Ownership of motorized transport	Own = Ref Not Own = 1.32 (1.17–1.49)	[25]
Obstetric/ Access to health information	Parity	Para 1;Ref para 2 = 0.61 (0.39–0.93) para 3+ = 0.31 (0.18–0.55)	[28]
		Para 0–1 = Ref Para 2 and above- AOR, 0.46, *P*-value< 0.05	[14]
	Receiving information on pregnancy issues from a health worker	Not received = Ref Received = 1.75 (1.51–2.02)	[25]
	Receiving support from family member	No support = Ref Support = 1.37 (1.19–1.59)	[25]

*Ref* Reference category, *Yrs* Years

### Characteristics of studies included in systematic review and meta-analysis of IPV and skilled delivery care utilization

Overall, seven studies with a total of 40,257 participants were included for skilled delivery care utilization assessment. Among the included studies, four  were from Africa [13, 15, 24, 30], and two from Asia [27, 28] while the remainder were multi-country studies from low and middle-income countries [29]; all were community-based studies. Regarding the types of IPV reported, five studies reported on physical violence [13, 24, 28–30], four studies on sexual violence [13, 24, 28, 30], and three studies on emotional violence [13, 24, 30] while combined IPV was reported in three studies [15, 27, 28]. Regarding IPV assessment tools, three studies used the DHS tool [13, 27, 29], another three studies used the conflict tactics scale [15, 28, 30] while the remaining study used the WHO tool [24].

Concerning the period of IPV ascertainment, three studies reported on IPV ever experienced by women in a current relationship [13, 24, 27], two studies reported on IPV experienced during lifetime [28, 29], one study reported on IPV experienced in the year prior to the survey being conducted [15] while one study reported on IPV experienced in any relationship since 15 years of age [30] (Table 4).

**Table 4:** Characteristics of studies included in the meta-analysis of IPV and skilled delivery care utilization

Study	Sample size	Study location	Study setting	Types of violence	IPV exposure time	IPV assessment scale	NOS quality score	Confounders
[13]	975	Kenya	Community /Kenya DHS	emotional, sexual, and physical IPV	Ever in current relationship	DHS tool	5	Women’s education, wealth index, residence, number of antenatal visits, and parity
[15]	858	Uganda	Community /Uganda DHS	physical and sexual IPV	Prior year	Conflict Tactic scale	5	Women’s education, economic empowerment of women, partner education, wealth index, number of children, ANC visit, women’s ability to negotiate condom /avoid sex.
[24]	210	Ethiopia	Community /Primary survey	Physical, Sexual, emotional, partner control	Ever in current relationship	WHO tool	6	women’s age, couple’s age gap, women’s educational status, partner’s educational status, women’s decision-making autonomy, women’s employment status, women’s weekly mass media exposure, couple’s relationship duration and household monthly income
[27]	294	Timor-Leste	Community /Timor-Leste DHS	IPV general	Ever in current relationship	DHS tool	4	Age of the women, women’s education, and wealth.
[29]	18,507	Multi country	Community/DHS	Physical	Lifetime	DHS tool	3	Women’s age, partner’s age, marital status, residency, house hold wealth index, women’s education, partner’s education, women’s having job, partner having job
[30]	17,412	Nigeria	Community / Nigeria DHS	Physical, sexual, emotional	Ever IPV in any relation since 15 years of age	Conflict Tactic scale	6	Women’s age, women’s education, husband’s education, employment status, women’s autonomy, parity, access to media, household wealth, household size, place of residency.
[28]	2001	Bangladesh	Community / Bangladesh DHS	Physical, sexual, combined IPV	Lifetime	Conflict tactic scale	5	Women’s age, women’s education, husband’s education, women’s decision-making and freedom of movement autonomy, women’s occupation, residence, religion, frequency of mass media exposure, parity, pregnancy intentions, and wealth index category

### Relationship between IPV and skilled delivery care utilization

**Fig. 4:**
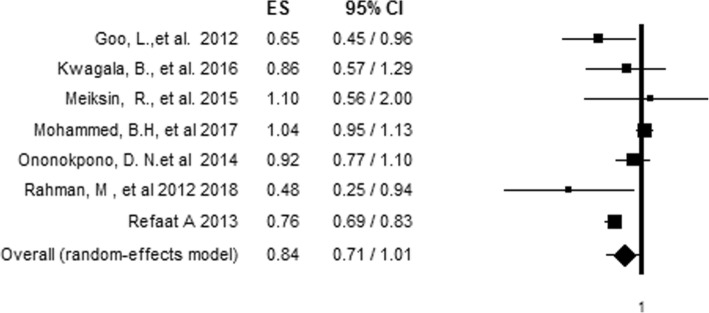
Forest plot of an association between IPV and skilled delivery care utilization before sensitivity analysis

**Fig. 5:**
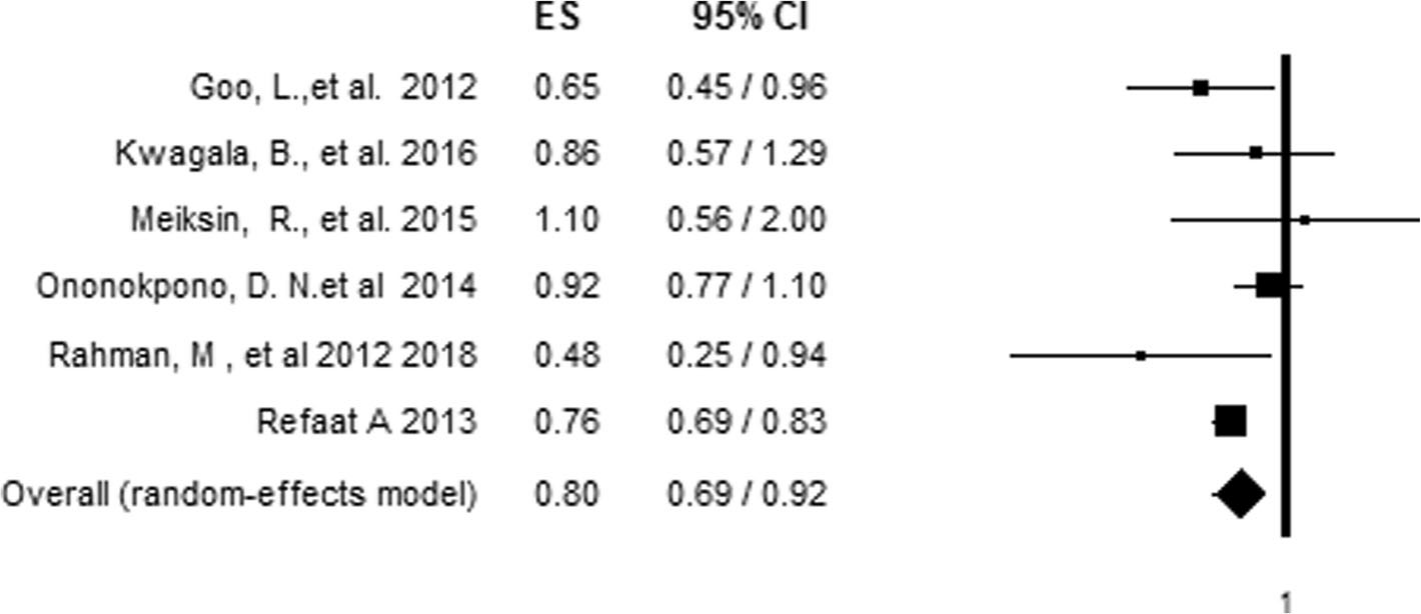
Forest plot of an association between IPV and skilled delivery care utilization after sensitivity analysis using the leave-one-out approach

To evaluate the relationship between IPV and antenatal care adequacy, articles that reported on more than one aspect of IPV in relation to antenatal care adequacy were first made to be represented by a single effect size, giving preference for combined IPV [28] and the effect size with the most precise estimate respectively [13, 24, 30]. The association of each aspect of IPV with skilled delivery care utilization was assessed by pooling the effect of each aspect of IPV in the analysis. The pooled analysis showed no significant association between overall IPV and skilled delivery care utilization (AOR= 0.84, 95%CI = 0.71, 1.01) (Fig. 4) indicating a high level of hetrogeniety (80.12%) among inclued studies with no significant risk of bias (Egger's test *P* value of 0.556) (Table 5).  However, after we conducted a sensitivity analysis by removing the most influential study [24] using the leave-one-out approach [31], heterogeneity fell to 36.36% with a pooled effect size indicating 20% reduced odds (AOR = 0.8, 95%CI = 0.69, 0.92) of using skilled delivery care among women who experienced IPV compared to those who did not experience IPV (Fig. 5).

**Table 5:** Hetrogeneity test for Meta-analysis of IPV and skilled delivery care utilization

Types of IPV	Hetrogeniety test				
	X^2^	P-value	I^2^	T^2^	T
Combined	3.37	0.186	40.60	0.06	0.24
Emotional	4.68	0.096	57.29	0.05	0.23
Physical	10.27	0.036	61.05	0.02	0.15
Sexual	4.57	0.102	56.21	0.03	0.16
Over all hetrogeniety before sensitivity analysis	30.18	0.001	80.12	0.03	0.18
Over all hetrogeniety after sensitivity analysis	7.86	0.164	36.36	0.01	0.10

### Different types IPV and skilled delivery care utilization

**Fig. 6:**
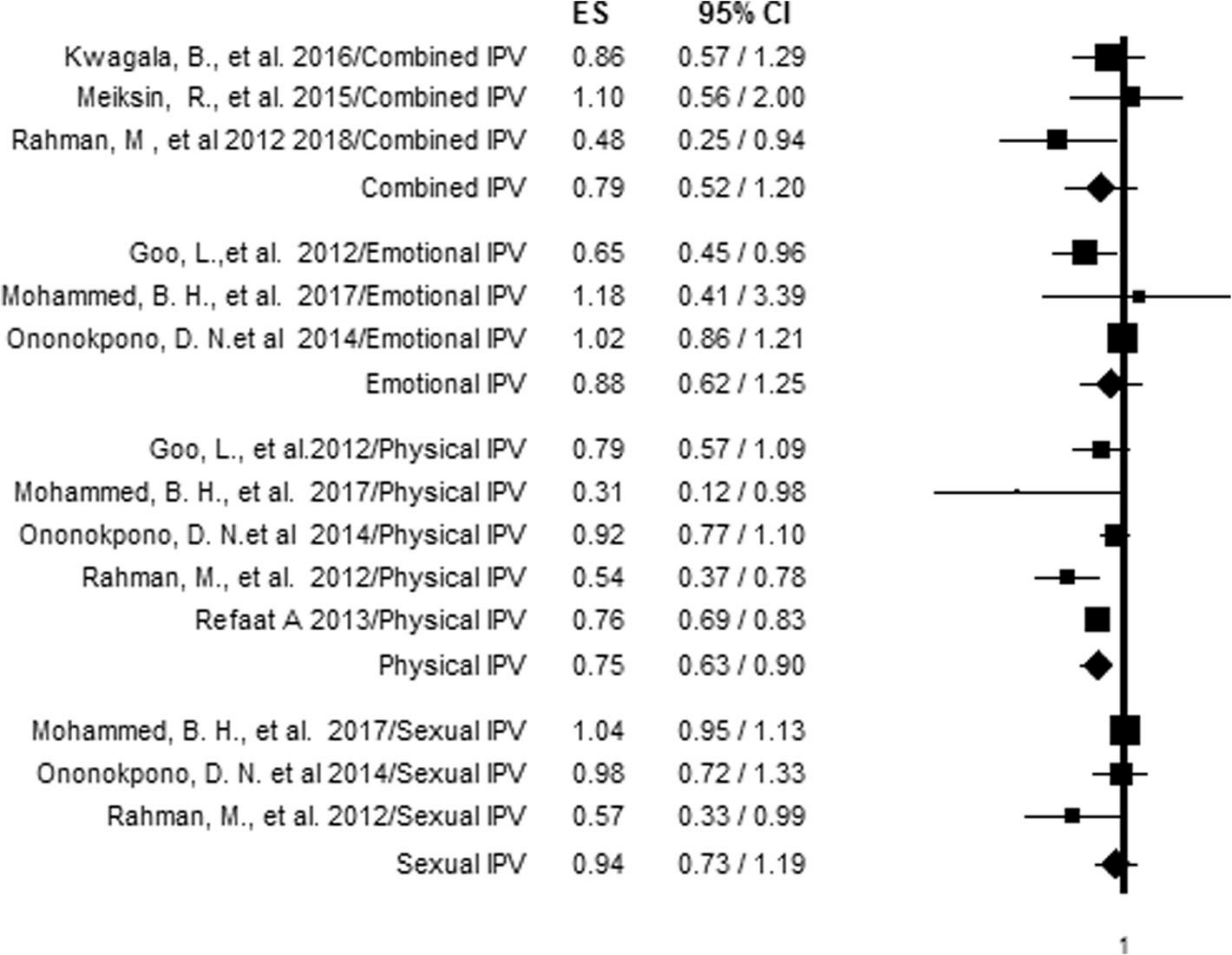
Forest plot of an association between different types of IPV and skilled delivery care utilization

Due to the existance of studies that reported on more than one aspect of IPV, we also carried out further analysis to precisely establish the relationship between each aspect of IPV and skilled delivery care utilization. Specific IPV types were included in the analysis if reported by two or more studies. Partner control was excluded from the analysis, as only one article reported it. From the analysis by type, only physical violence was associated with using skilled delivery care. Women who experienced physical violence had 25% decreased odds (AOR = 0.75, 95%CI = 0.63, 0.90) of using skilled delivery care compared to those who did not experience physical IPV (Fig. 6). Furthermore, the levels of heterogeneity among the studies were not significant, except for physical and emotional violence, which indicated heterogeneity levels of 61.05 and 57.29% respectively (Table 5).

### Factors associated with utilizing skilled delivery care

The reviewed studies indicated an association between skilled delivery care utilization and women’s age. In two studies which investigated this association, it was shown that women aged 15–24 were less likely to receive skilled delivery compared to those above 24 years of age [28, 30]. Women who were married were more likely to utilize skilled delivery care compared to unmarried women [29]. Similarly, women who had received primary and above education [29, 30] and whose partner had received primary and above education [15, 28–30] were more likely to use skilled delivery care. Women with a DHS wealth index categorized as rich were more likely to utilize skilled delivery care [29, 30]. Moreover, being from an urban area was associated with increased utilization of skilled delivery care compared to living in a rural area [27–30]. In one study, an association was reported between being in work and utilizing skilled delivery care, indicating women who had work were more likely to utilize skilled delivery care [30].

The review showed an association between multiparity and skilled delivery care use. Women who were multiparous were less likely to utilize skilled delivery care [15, 30]. Similarly, having four or more antenatal care visits was associated with an increased likelihood of using skilled delivery care [15]. In addition, having access to newspapers, TV and radio was associated with utilizing skilled delivery care [28] (Table 6).

**Table 6:** Covariates associated with skilled delivery care utilization

covariate	Variables	Skilled delivery care utilization	
		Reported AOR, 95% CI	Reference
Socio-demographic	Age	15–24 yrs. = Ref 25-34yrs = 1.23 (1.05,1.45), 35–49 yrs. = 1.45 (1.16–1.80)	[30]
		15–24 yrs. = Ref, 25–34 yrs. = 2.07 (1.22–3.49) 35–49 yrs. = 4.08 (1.95–8.54)	[28]
	Maternal education	No education = Ref Primary = 1.43 (1.20,1.69), Secondary+ = 2.89 (1.39,3.50)	[30]
		No education = Ref, Primary = 2.33 (1.03,5.27), Secondary+ = 5.40 (2.45,11.87)	[28]
		Uneducated = Ref Educated = 3.08 (2.72–3.49)	[29]
	Husband’s Education	No education = Ref Primary = 1.46 (1.22, 1.75), Secondary+ = 1.81 (1.51, 2.17).	[30]
		No education = Ref Secondary+ = 2.3 [1.18–4.50]	[15]
		No education = Ref Secondary+ = 1.80 (1.05–3.08).	[28]
	Employment status	Not working = Ref. Working 1.24 (1.09–1.42).	[30]
	Wealth index	Poor = Ref, Middle = 2.02 (1.77–2.31) Rich = 2.89 (2.40–3.47)	[30]
		Poor = Ref Rich = 1.27 (1.13–2.88)	[28]
		Poorest = Ref Rich = 2.59 [1.41–4.76]	[15]
		Poor = Ref Rich = 2.47 (2.22–275)	[29]
	Residency	Urban = Ref, Rural = 0.69 (0.60–0.79).	[30]
		Rural = Ref Urban = 3.67 (3.29–4.09)	[29]
		Rural = Ref Urban = 1.88 (1.18–3.01).	[28]
	Marital status	Unmarried/not in union = Ref Married/in union = 1.45 (1.01–1.30)	[29]
Obstetric/ Access to health information	Parity	para 1 = Ref para 2–3, 0.66 (0.56–0.79) para 4+, 0.59 (0.46–0.75)	[30]
		Para 1 = Ref, Para 2–4 = 0.40 [0.22–0.74] Para 5 + =0.40 [0.21–0.75]	[15]
	ANC	< 4 ANC visits = Ref 4+ ANC = 1.93 [1.34–2.79]	[15]
	Access to Newspaper	No access = Ref Access = 1.84 (1.36–2.48),	[30]
	Access to TV	No Access = Ref Accesses = 1.53 (1.36–1.71)	[30]
	Access to Radio	No access = Ref Access = 1.02 (0.84–1.23)	[30]

*Ref* Reference category, *Yrs* Years

## Discussion

Our meta-analyses have demonstrated an association between IPV and maternal health care services (antenatal care and skilled delivery care) utilization. Women who experienced IPV had 25% decreased odds (AOR = 0.75, 95%CI = 0.61, 0.92) of using adequate antenatal care compared to those who did not experience IPV. Similarly, women who experienced IPV had 20% decreased odds (AOR = 0.8, 95%CI = 0.69, 0.92) of using skilled delivery care compared to those who did not experience IPV. This may be due to the potential impact of IPV on women’s ability to access health care services through limited decision-making power, reduced freedom of movement and higher economic dependency [32, 33].

We conducted further analysis to precisely establish the relationship between each aspect of IPV and maternal health care services (antenatal care and skilled delivery care) utilization. The analysis indicated that experiencing physical IPV and combined IPV was associated with inadequate utilization of antenatal care whereas experiencing only physical IPV was associated with decreased odds of using skilled delivery care.

Women who experience physical violence that result in injury may refrain from attending a health facility, so as not to expose the evidence of IPV to a third person due to feelings of shame, embarrassment or fear of repercussions [34]. In addition, this might hypothesize pathways linking IPV experience to poor maternal health care usage through long-term mental health effects, such as anxiety or depression, which might reduce women’s desire to obtain health care services [35, 36]. Furthermore, the association between combined IPV and not receiving adequate antenatal care indicated the increased risk of multiple IPV exposures in impeding utilization of adequate antenatal care services.

In a further analysis of IPV types and skilled delivery care utilization, only experiencing physical violence was found to be associated with utilizing skilled delivery care, while experiencing combined, emotional and sexual IPV showed no association with skilled delivery care utilization. This may be the result of the small number of studies included in the meta-analysis examining combined, emotional and sexual IPV, as previous evidence has indicated combining small numbers of studies may result in reduced statistical power [37].

In addition to the meta-analysis, we also reviewed other possible factors associated with receiving adequate antenatal care and skilled delivery care that were reported in the included studies. Socio-demographic and reproductive health related factors, including having an educated partner [25, 28, 30], being younger [25, 30], living in an urban area [28, 30], being rich [28, 30] and being high parity [28, 30] were associated with receiving antenatal care and skilled delivery care. Similar systematic reviews of studies have indicated the effect of socio-demographic and obstetric characteristics on utilization of maternal health care services, including antenatal care and skilled delivery care [38, 39] indicating the importance of addressing socio-demographic and reproductive factors in improving maternal health care service uptake. Although the review showed possible factors that need to be addressed to improve maternal health care services, a limited number of articles included in the study indicate the existence of research gaps and the need for further research to increase understanding of the complex interaction of IPV with other factors that affect the use of maternal health care services.

### Strengths and limitations of the study

This meta-analysis is the first of its kind to analyse the existing evidence to establish the associations between IPV and maternal health service (antenatal care and skilled delivery care) utilization. One of the major strengths of this study was the use of a sensitive and thorough search strategy to include a large number of studies without limiting the search by date of publication and geographical region. Although this analysis indicated the association of IPV with outcome variables, it has its own shortcomings. One weakness of the study was that all studies included were cross-sectional in design, which precludes temporal or causal modelling. This indicates the importance of conducting further studies in this area by using stronger longitudinal or cohort designs.

In addition, the existence of heterogeneity among studies included in the analysis was observed and reducing further heterogeneity was not possible using a subgroup analysis, as a subgroup analysis requires a large volume of individual studies to make meaningful interpretations from the data [40]. However, there is no consensus regarding the level of heterogeneity that should be reported in a meta-analysis study; some of the studies reported a 99.1% I-squared level of heterogeneity [41]. Consistent with this, other researchers suggest that it is valid to report findings even with heterogeneity as long as the predefined eligibility criteria for meta-analysis are sound and the data are correct [42].

The findings of the study might not necessarily reflect the relationship of IPV specific to pregnancy and maternal health care services utilization, as studies included in the meta-analysis reported different timing in exposure to IPV, including lifetime IPV. In addition, caution should be taken in interpreting the findings presented herein in the context of the developed world, as almost all studies included in the meta-analysis were from the developing world. The exclusion of studies published in a language other than English might introduce bias. Apart from its limitations, the present review provided a base for evidence on the effect of IPV on maternal health care services use and indicated the need for urgent action to prevent IPV.

## Conclusion

IPV is associated with a lower likelihood of receiving adequate antenatal care and skilled delivery care. Both community-based and facility-based interventions that target the reduction of IPV [43], such as facility-based counselling interventions [44], might aid in improving the utilization of maternal health care services. As all the included studies were cross-sectional, further research needs to be done using longitudinal studies in order to generate accurate evidence.

## Additional file

Additional file 1: Search summary of systematic review and Meta -analysis of association of IPV with antenatal care and skilled delivery care utilization. (DOCX 13 kb)

## Abbreviations

DHS: Demographic and Health Survey
IPV: Intimate Partner Violence
NOS: Newcastle-Ottawa Scale
WHO: World Health Organization
