# Supplementary material for: The associations between intimate partner violence and maternal health care service utilization: a systematic review and meta-analysis
Source: BMC Womens Health. 2019 Feb 26;19:36. doi: 10.1186/s12905-019-0735-0 (PMC6390526; doi:10.1186/s12905-019-0735-0)
Supplement: Supplementary file 1 — Search summary of systematic review and Meta -analysis of association of IPV with antenatal care and skilled delivery care utilization. (DOCX 13 kb) [file 12905_2019_735_MOESM1_ESM.docx]

**Supplementary file 1: Search summary of Systematic review and Meta -analysis of association of IPV with ANC and SDC utilization.**

| **Data base searched** | **Date searched** | **Search Term 1** | **Search tem 2** |
| --- | --- | --- | --- |
| Medline | 05/09/2017 | Battered Women/ or Spouse Abuse/ or Intimate Partner Violence/ or Domestic Violence/ OR partner abuse.mp. OR ((spouse* or Partner* or Domestic or famil* or Wife or wives) adj3 (Violen* or Assault* or abuse)).tw. | Antenatal care.mp. or Prenatal Care/ OR maternal health care*.mp. or Delivery, Obstetric/ OR Delivery at health facilit*.mp. OR Delivery, Obstetric/ or Skilled birth attendan*.mp.OR maternal care service*.mp. OR ((deliver* or birth*) adj10 (institution* or facilit* or center* or centre* or hospital* or clinic*)).mp. OR pregnan*.tw. OR Maternal Health Services/ |
| EMBASE | 05/09/2017 | Battered Women/ or Spouse Abuse/ or partner violence.mp. or Domestic Violence/ OR Intimate Partner Violence/ OR partner abuse.mp OR ((spouse* or Partner* or Domestic or famil* or wife or wives) adj3 (Violen* or Assault* or abuse)).tw | Antenatal care.mp. or Prenatal Care/ OR Prenatal Care/ or maternal health care.mp. or Delivery, Obstetric/ OR maternal care service*.mp. OR Delivery at health facilit*.mp. or Delivery, Obstetric/ OR Obstetrics/ or institutional delivery.mp. OR Skilled birth attendan*.mp OR maternal health care*.mp. OR maternal health service*.mp.OR pregnan*.tw. OR ((deliver* or birth*) adj10 (institution* or facilit* or center* or centre* or hospital* or clinic*)).mp. |
| PsychINFO | 05/09/2017 | Partner Abuse/ or Intimate Partner Violence/ or Domestic Violence/ or Partner violence.mp. OR battered females.mp. OR spouse abuse.mp. OR ((spouse* or Partner* or Domestic or famil* or wife or wives) adj3 (Violen* or Assault* or abuse)).tw | exp Prenatal Care/ or exp Pregnancy/ or Antenatal care.mp.OR exp Birth/ or exp Obstetrics/ or Skilled birth attendant.mp. OR exp Health Care Utilization/ or exp Health Care Services/ or exp Health Care Seeking Behavior/ or maternal health care.mp. OR maternal care service*.mp. OR Birth/ or Delivery at health facilit*.mp. OR ((deliver* or birth*) adj10 (institution* or facilit* or center* or centre* or hospital* or clinic*)).mp. OR maternal health care*.mp. OR maternal health services.mp. OR pregnan*.tw. |
| Maternity and Infant care | 05/09/2017 | Domestic violence.de. or Partner violence.mp. OR partner abuse.mp. OR Battered women.mp. OR ((spouse* or Partner* or Domestic or famil* or wife or wives) adj3 (Violen* or Assault* or abuse)).tw.OR Intimate partner violence.mp. OR spouse abuse.mp. | (Maternal health services or Maternal health).de.OR maternal care service*.mp. OR Delivery at health facilit*.mp. OR Skilled birth attendan*.mp. OR prenatal care.mp. spouse abuse.mp. OR institutional delivery.mp. OR ((deliver* or birth*) adj10 (institution* or facilit* or center* or centre* or hospital* or clinic*)).mp. OR pregnan*.tw. |
| CINAHL | 05/09/2017 | ((spouse* or Partner* or Domestic or famil* or wife or wives) adj3 (Violen* or Assault* or abuse)).tw. OR (MH "Domestic Violence") OR "partner abuse" OR "spouse abuse" OR (MH "Battered Women") OR (MH "Intimate Partner Violence") | pregnan*tw. OR ((deliver* or birth*) adj10 (institution* or facilit* or center* or centre* or hospital* or clinic*)).mp. OR skilled birth attendan* OR "delivery at health facilit*" OR (MH "Delivery, Obstetric") OR maternal care service* OR (MH "Maternal Health Services") OR "maternal health care*" OR (MH "Prenatal Care") OR "antenatal care" |
